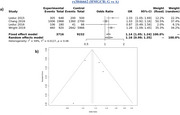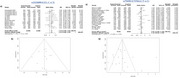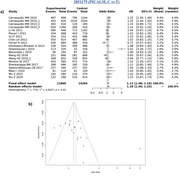# Polymorphisms in brain cholesterol homeostasis pathway as biomarkers for Alzheimer's Disease: meta‐analyses of clinical evidences

**DOI:** 10.1002/alz70856_105361

**Published:** 2026-01-07

**Authors:** PRAISY K PRABHA, Ashish Jain, Niharika Dadoo, Shiv Charan, Bikash Medhi, Ajay Prakash

**Affiliations:** ^1^ Postgraduate Institute of Medical Education & Research (PGIMER), Chandigarh, Chandigarh, India; ^2^ Postgraduate Institute of Medical Education and Research (PGIMER), Chandigarh, Chandigarh, India

## Abstract

**Background:**

Alzheimer's disease (AD) is a neurodegenerative condition marked by memory loss, cognitive decline, and eventually motor and behavioural dysfunction. Most AD drug trials have failed due to the lack of early intervention, which is crucial for treatment effectiveness. Though early diagnosis remains challenging owing to blood‐brain barrier, blood‐based biomarkers are being explored due to their non‐invasive nature. Genes involved in cholesterol and lipid metabolism, such as APOE, APOJ, ABCA7, and SORL1, have also been observed to increase AD risk.

**Methods:**

Clinical studies of polymorphisms in cholesterol homeostasis pathway involving participants clinically diagnosed with Alzheimer's Disease of any form as per set criteria of diagnosis for AD were included after comprehensive search across PubMed, Embase, Scopus and Web of Science. Independent reviewers extracted data from the included studies which included information like general information, participants, study methods, polymorphisms studied, outcomes, results, conclusion, etc. Any discrepancies or doubts was resolved by a third reviewer.

**Result:**

A total of 1870 studies were identified based on the designed search strategy, which reduced to 216 after removal of duplicates, with 45 studies considered suitable for the final meta‐analyses. The risk of AD was significantly associated in random effect model for SNP rs3846662 (HMGCR; OR = 1.16, 95% CI = 0.99, 1.35, I^2^ = 59%, *p* = 0.06), rs11136000 (CLU; OR = 1.15, 95% CI = 1.08, 1.22, I^2^ = 0%, *p* = 0.83), rs754203 (CYP46A1; OR = 1.10, 95% CI = 0.92, 1.33, I^2^ = 87%, *p* < 0.01), and rs3851179 (PICALM; OR = 1.18, 95% CI = 1.05, 1.33, I^2^ = 77%, *p* < 0.01).

**Conclusion:**

The selected SNPs were found to be significantly associated with the risk of AD, with risk alleles for rs3846662, rs11136000, rs754203, and rs3851179 being G, C, T, C alleles respectively with an OR of 1.16, 1.15, 1.10, and 1.18 respectively. Therefore, these can be considered to be AD biomarkers.